# A systematic review: Cost‐effectiveness of continuous glucose monitoring compared to self‐monitoring of blood glucose in type 1 diabetes

**DOI:** 10.1002/edm2.369

**Published:** 2022-09-16

**Authors:** Yuxin Jiao, Rose Lin, Xinyang Hua, Leonid Churilov, Michele J. Gaca, Steven James, Philip M. Clarke, David O'Neal, Elif I. Ekinci

**Affiliations:** ^1^ Austin Health Heidelberg Victoria Australia; ^2^ Centre for Health Policy Melbourne School of Population and Global Health University of Melbourne Carlton Victoria Australia; ^3^ Melbourne Medical School The University of Melbourne Parkville Victoria Australia; ^4^ Health Sciences Library Austin Health Heidelberg Victoria Australia; ^5^ School of Nursing, Midwifery and Paramedicine University of the Sunshine Coast Petrie Queensland Australia; ^6^ Health Economics Research Centre Nuffield Department of Population Health University of Oxford Oxford UK; ^7^ Department of Medicine St Vincent's Hospital Melbourne, Melbourne Medical School, The University of Melbourne Parkville Victoria Australia; ^8^ Department of Medicine, Austin Health Melbourne Medical School, The University of Melbourne Parkville Victoria Australia; ^9^ Department of Endocrinology Austin Health Heidelberg Victoria Australia

**Keywords:** continuous glucose monitoring, cost‐analysis, cost‐effectiveness, health economics, self‐monitoring blood glucose, type 1 diabetes

## Abstract

Continuous glucose monitoring (CGM) is rapidly becoming a vital tool in the management of type 1 diabetes. Its use has been shown to improve glycaemic management and reduce the risk of hypoglycaemic events. The cost of CGM remains a barrier to its widespread application. We aimed to identify and synthesize evidence about the cost‐effectiveness of utilizing CGM in patients with type 1 diabetes. Studies were identified from MEDLINE, Embase and Cochrane Library from January 2010 to February 2022. Those that assessed the cost‐effectiveness of CGM compared to self‐monitored blood glucose (SMBG) in patients with type 1 diabetes and reported lifetime incremental cost‐effectiveness ratio (ICER) were included. Studies on critically ill or pregnant patients were excluded. Nineteen studies were identified. Most studies compared continuous subcutaneous insulin infusion and SMBG to a sensor‐augmented pump (SAP). The estimated ICER range was [$18,734–$99,941] and the quality‐adjusted life year (QALY) gain range was [0.76–2.99]. Use in patients with suboptimal management or greater hypoglycaemic risk revealed more homogenous results and lower ICERs. Limited studies assessed CGM in the context of multiple daily injections (MDI) (*n* = 4), MDI and SMBG versus SAP (*n* = 2) and three studies included hybrid closed‐loop systems. Most studies (*n* = 17) concluded that CGM is a cost‐effective tool. This systematic review suggests that CGM appears to be a cost‐effective tool for individuals with type 1 diabetes. Cost‐effectiveness is driven by reducing short‐ and long‐term complications. Use in patients with suboptimal management or at risk of severe hypoglycaemia is most cost‐effective.

## Graphical abstract

Continuous glucose monitoring has advanced significantly in the last few years. Multiple studies and meta‐analysis have reported the benefit of continuous glucose monitoring in improving diabetes management and hypoglycemic risk. Cost has however remained a barrier to its widespread use. This review summarises available studies assessing the cost‐effectiveness of CGM.

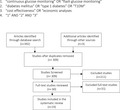

## 1 INTRODUCTION

With the exponential growth of technology, our ability to support individuals with type 1 diabetes has improved dramatically. One pivotal technology that has and will continue to help achieve this is continuous glucose monitoring (CGM). With each passing year, the clinical benefits of CGM have become more pronounced. In the long term, integrating CGM into standard diabetes care will play a crucial part in determining our future success in preventing diabetes‐related complications. Though the benefits of CGM are evident, cost remains a significant barrier to its widespread use. To expedite the use of CGM in the type 1 diabetes population, appropriate funding will be required and thus economic evaluation of CGM is vital for informed policymaking.

Diabetes‐related complications remain a significant burden for individuals with type 1 diabetes. Historically, to combat the morbidity of chronic hyperglycaemia, intensive treatment with lower HbA1c targets were utilized. Although this strategy reduced rates of chronic hyperglycaemic complications, it placed individuals with type 1 diabetes at a greater risk of hypoglycaemic events.^1^ With CGM there is evidence that chronic hyperglycaemia and hypoglycaemic events can both be avoided. For example, a recent trial showed that in older adults with type 1 diabetes, CGM was able to significantly reduce time spent in hypoglycaemia.^2^ Concurrently, studies have shown CGM reduces chronic hyperglycaemia with a significant reduction in HbA1c.^3^, ^4^ These improvements in physiological parameters have also translated into psychological benefits. With CGM use, individuals have reported reductions in diabetes‐related distress, improved hypoglycaemic confidence, and improvements in fear of hypoglycaemia (FoH) scores.^5^ Whilst these clinical improvements with CGM use are important for the individual, benefits also extend to wider society due to the reduction in acute service use over time.

The importance of reducing diabetes‐related complications extends beyond the individual, as diabetes‐related complications also represent a significant economic burden on all healthcare systems. Hypoglycaemia alone is estimated to be responsible for 100,000 emergency department visits in the United States of America (USA)^6^; with a total annual estimated cost between USD 1.8 to 5.9 billion.^7^ This is on top of the cost associated with macro‐ and microvascular injury from chronic hyperglycaemia. The American Diabetes Association reported cardiovascular complications, for people with both type 1 and type 2 diabetes, were responsible for 27% of the total cost of treating diabetes in the USA.^8^ This equates to USD 37.3 billion spent on diabetes associated cardiovascular disease.^8^ Thus, there is hope that CGM usage costs will be offset by the reduction in diabetes‐complication related expenditure. This will be further compounded by advancements in CGM, which has already seen the cost of the technology decline. Given the chronic nature of diabetes and, the rapid evolution of CGM, timely evaluation of CGM cost‐effectiveness relies on statistical modelling rather than long‐term observation studies. Many studies have attempted to assess the lifetime cost‐effectiveness of CGM through simulation modelling. The current study aims to summarize and clarify the findings of these studies, to provide guidance in the appropriateness of implementing CGM technology. Our hypothesis is that CGM is a cost‐effective diabetes management tool through reducing complication costs and improving the quality of life for individuals with type 1 diabetes.

## 2 METHODS

### 2.1 Inclusion and exclusion criteria

In this systematic review, we included studies that assessed the cost‐effectiveness of using CGM compared to self‐monitored blood glucose (SMBG) in monitoring blood glucose levels in patients with type 1 diabetes, regardless of the mode of insulin delivery. Studies were retrospective in nature and were required to report a lifetime ICER as an outcome. They were also required to focus on modelling for an adult population. Studies were excluded if they focused on a specific cohort of patients, to allow for more generalisable results.

### 2.2 Search strategy

**FIGURE 1:**
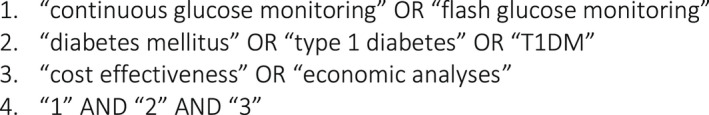
Search strategy

A search was conducted of the MEDLINE, Embase and the Cochrane Library databases for the period between January 2010 to February 2022, with no country limits applied. A date limit of 2010 onwards was applied due to the rapid improvements in technology, so outcomes are better matched to the technology used today. Our search strategy utilized Medical Subject Headings and text words related to “continuous glucose monitoring”, “flash glucose monitoring”, “type 1 diabetes”, “cost effectiveness” and “economic analyses” as described in Figure 1. The database searches were complimented by grey literature searches using Google's Advanced Search. To ensure literature saturation, we reviewed the reference lists of included studies or relevant reviews identified.

### 2.3 Method of review

**FIGURE 2:**
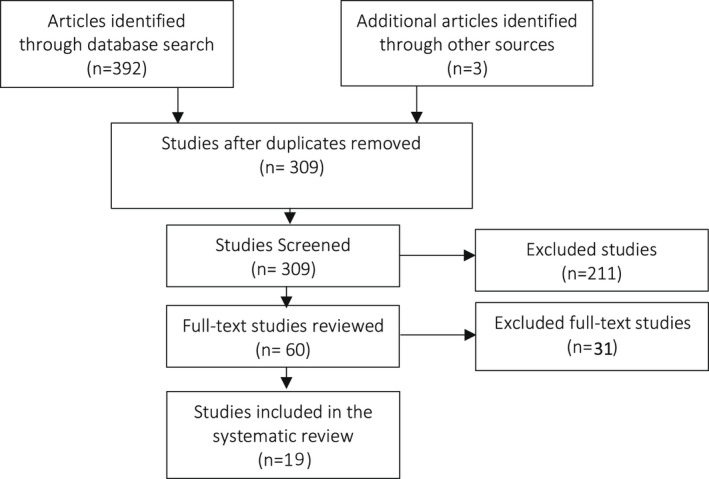
Systematic review flow diagram

All studies identified were collated into an EndNote database and deduplicated before being uploaded to Covidence for screening. Through Covidence, two reviewers independently screened abstracts to identify articles potentially meeting the inclusion criteria. For those articles, full text versions were retrieved and once again independently screened by the two reviewers to determine whether they met inclusion criteria. Any disagreements about whether the inclusion criteria were met were resolved through discussion between the two reviewers. If no consensus could be reached a third reviewer was utilized to make a final decision. Review process summarized in Figure 2.

### 2.4 Data extraction

Data extraction of relevant study information for articles meeting inclusion criteria was performed independently by two reviewers. Disagreements were resolved through discussion and if required a third reviewer's input. A data extraction template was created through Covidence to collect relevant information regarding the study design, location, intervention used, study population, calculation, source of inputs and main findings.

### 2.5 Statistical modelling

Multiple simulation models have been built to predict the progression of diabetes and its complications. Many of these models utilize Markov modelling to predict progression by simulating movement between different health states in a cohort of individuals. In the current context, these health states represent the development of diabetes‐related complications. Outcomes in the simulated scenarios are based on various parameters encapsulating underlying assumed probability distributions. Costs are also assigned to each complication. An ICER can be calculated by the difference in cost accumulated between two groups (control/intervention) divided by the difference in the quality‐adjusted life years (QALY). Because there are no agreed methods for pooling estimates of cost effectiveness, we did not conduct meta‐analysis of the cost‐effectiveness results.^9^

### 2.6 Currency conversion

To make meaningful comparisons between the studies ICER were expressed using a common currency. Extracted ICERs were converted into 2021 AUD using the Campbell and Cochrane Economics Methods Group tool as recommended by Cochrane.^10^, ^11^ The conversion tool adjusts for estimates of costs for currency and price year. All costs depicted in this paper are in AUD unless otherwise specified.

## 3 RESULTS

### 3.1 Description of studies

The initial search identified 311 citations accepted for screening and complete abstract review. Of these initial abstracts, 98 citations were deemed to potentially meet the inclusion criteria. Additionally, three articles were identified when scanning the references of relevant published articles in the field. After review of the full text 19 studies from 11 countries were included. Common reasons for exclusion included focus on type 2 diabetes and lack of ICER as a primary or secondary outcome.

### 3.2 Description of study features

Study characteristics are detailed in Table 1. All 19 studies utilized Markov modelling to calculate ICER, including 12 studies which used various versions of the CORE diabetes model. These studies assessed cost‐effectiveness from the viewpoint of 11 countries. With regards to the associated treatment, seven studies compared continuous subcutaneous insulin infusion (CSII) and SMBG versus sensor‐augmented pump (SAP).^12^, ^13^, ^14^, ^15^, ^16^, ^17^, ^18^, ^25^ Four studies^19^, ^20^, ^21^, ^22^ assessed CGM versus SMBG in the context of multiple daily injections (MDI); two studies compared MDI and SMBG with SAP^23^, ^24^; three studies assessed SMBG with MDI or CSII in comparison with hybrid closed‐loop systems (HCL)^25^, ^26^, ^27^ and three studies^28^, ^29^, ^30^ compared CGM and SMBG regardless of the mode of insulin delivery. Fifteen of the articles were funded by medical device companies, one article was funded by the Juvenile Diabetes Research Foundation Grant and three received no funding.

**TABLE 1:** Study characteristics

	Study	Study model	Treatment	Funding
Roze et al. (2015)^12^	Health‐economic analysis of real‐time continuous glucose monitoring in people with Type 1 diabetes	CORE diabetes model	CSII only vs SAP	Medtronic funding
Roze et al. (2016)^13^	Cost‐effectiveness of sensor‐augmented pump therapy with low glucose suspend versus standard insulin pump therapy in two different patient populations with type 1 diabetes in france	CORE diabetes model	CSII only vs. SAP	Medtronic funding
Roze et al. (2016)^14^	Long‐term health economic benefits of sensor‐augmented pump therapy vs. continuous subcutaneous insulin infusion alone in type 1 diabetes: a U.K. perspective	CORE diabetes model	CSII only vs. SAP	Medtronic funding
Roze et al. (2017)^15^	Cost‐effectiveness of sensor‐augmented pump therapy versus standard insulin pump therapy in patients with type 1 diabetes in Denmark	CORE diabetes model	CSII only vs. SAP	Medtronic funding
Conget et al. (2018)^16^	Cost‐effectiveness analysis of sensor‐augmented pump therapy with low glucose‐suspend in patients with type 1 diabetes mellitus and high risk of hypoglycaemia in Spain	IQVIA CORE diabetes model	CSII only vs. SAP	Medtronic funding
Nicolucci et al. (2018)^17^	Cost‐effectiveness of sensor‐augmented pump therapy in two different patient populations with type 1 diabetes in Italy	CORE diabetes model	CSII only vs. SAP	Medtronic funding
Roze et al. (2019)^18^	Cost‐effectiveness of sensor‐augmented insulin pump therapy versus continuous insulin infusion in patients with type 1 diabetes in Turkey	IQVIA CORE diabetes model	CSII only vs. SAP	Medtronic funding
Chaugule et al. (2017)^19^	Cost‐effectiveness of G5 Mobile continuous glucose monitoring device compared to self‐monitoring of blood glucose alone for people with type 1 diabetes from the Canadian societal perspective	IMS CORE diabetes model (v.9.0) (cohort‐based bootstrap model)	MDI for both	Dexcom funding
Wan et al. (2018)^20^	Cost‐effectiveness of continuous glucose monitoring for adults with type 1 diabetes compared with self‐monitoring of blood glucose: The DIAMOND randomized trial	Sheffield diabetes model	MDI for both	Dexcom funding
Roze et al. (2020)^21^	Long‐term cost‐effectiveness of Dexcom G6 real‐time continuous glucose monitoring versus self‐monitoring of blood glucose in patients with type 1 diabetes in the UK	IQVIA CORE diabetes model	MDI for both	Dexcom funding
Roze et al (2021)^22^	Evaluation of the long‐term cost‐effectiveness of the Dexcom G6 continuous glucose monitor versus self‐monitoring of blood glucose in people with type 1 diabetes in Canada	IQVIA CORE diabetes model	MDI for both	Dexcom funding
Kamble et al. (2012)^23^	Cost‐effectiveness of sensor‐augmented pump therapy in adults with type 1 diabetes in the United States	CORE diabetes model	MDI vs. SAP	Medtronic and Duke University funding
Gomez et al. (2016)^24^	Clinical and economic benefits of integrated pump/CGM technology therapy in patients with type 1 diabetes in Colombia	IMS CORE Diabetes Model (CDM) version 8.5	MDI vs. SAP	Medtronic funding
Jendle et al. (2019)^25^	Cost‐effectiveness analysis of the MiniMed 670G hybrid closed‐loop system versus continuous subcutaneous insulin infusion for treatment of type 1 diabetes	IQVIA CORE diabetes model	CSII only vs. HCL	Medtronic funding
Pease et al. (2020)^26^	Cost‐effectiveness analysis of a hybrid closed‐loop system versus multiple daily injections and capillary glucose testing for adults with type 1 diabetes	Study's own Markov modelling	MDI vs. HCL	Nil
Roze et al (2021)^27^	Cost‐effectiveness of a novel hybrid closed‐loop system compared with continuous subcutaneous insulin infusion in people with type 1 diabetes in the UK	IQVIA CORE diabetes model	CSII only vs. HCL	Medtronic funding
Huang et al. (2010)^28^	The cost‐effectiveness of continuous glucose monitoring in type 1 diabetes	Study's own Markov modelling (Monte‐Carlo based)	(CSII 90% and MDI 10%)	Juvenile Diabetes Research Foundation grant
McQueen et al. (2011)^29^	Cost‐effectiveness of continuous glucose monitoring and intensive insulin therapy for type 1 diabetes	Study's own Markov Model w/input from CDC Cost‐Effectiveness Group model	CSII or MDI (varied)	Nil
Garcia‐Lorenzo et al. (2018)^30^	Cost‐effectiveness analysis of real‐time continuous monitoring glucose compared to self‐monitoring of blood glucose for diabetes mellitus in Spain	Study's own Markov Model	CSII and MDI	Nil

Baseline characteristics are provided in Table 2. Six studies assessed a specific subset of patients: (1) patients with suboptimal glycaemic management at baseline; (2) those who are at high risk of hypoglycaemia episodes; and (3) those with HbA1c <7%^28^ or (4) those aged >25 years.^21^ Table 2. also outlines the assumed clinical benefits of CGM compared to SMBG, as well as the source of these inputs. Input relating to HbA1c reduction were sourced from nine different studies and had results that ranged from 0.23% to 1.5% reduction. In one study the source of HbA1C reduction could not be identified.^16^ FoH score improvements ranged from 2.3 to 9.25‐points with use of CGM compared to SMBG. There was significant variability in the way reduction in hypoglycaemic events were reported.

**TABLE 2:** Baseline characteristics and treatment effects

Study	Age (years)	Baseline HbA1c (%)	Duration of diabetes (years)	Sex, female (%)	HbA1c % reduction	Rates of hypoglycaemia (SHE) per 100 patient‐years
Roze et al. (2015)^12^	27	8.60	13	54.5	SAP: 0.30	SAP vs Control: 23% reduction
Roze et al. (2016)^13^	36 ± 13.6^a^	9 ± 0.9	17 ± 10.8	47.0	SAP: 0.88 Control: 0.48*	Nil change
	18.6 ± 11	7.5 (7.2–7.9)	12 ± 8.9	50.0	Nil effect	CSII: 2.2 SAP: 0
Roze et al. (2016)^14^	27.1^**^	10	13	51.5	SAP: 1.49 Control: 0.62	CSII: 2.2 SAP: 0
Roze et al. (2017)^15^	27^a^	8.1	13.2	51.5	SAP: 0.56 Control: 0.13*	Nil change
	18.6^b^	7.5	11	50.5	Nil effect	CSII: 2.2 SAP: 0
Conget et al. (2018)^16^	18.6 ± 11.1	7.5 (7.2–7.9)	12 ± 8.9	50%	N/A	CSII: 2.2 events SAP: 0
Nicolucci et al. (2018)^17^	27 ± 15.6^a^	8.1 (1.3)	13.2 (10.8)	51.5	SAP: 0.56	Nil change
	SAP 19.7 ± 12.9^b^ CSII 17.4 ± 10.6	SAP 7.4 (7.2–7.6) CSII 7.6 (7.4–7.9)	SAP 12.1 (10.0) CSII 9.8 (7.4)	SAP 57.1 CSII 43.5	Nil Effect	SAP: 0 CSII: 2.2
Roze et al. (2019)^18^	27 ± 15.6^a^	9.0	13.2 ± 10.8	51.5	SAP: 1.1 Control: 0.36	Nil effect
	18.6 ± 11.8^b^	7.5	11 ± 8.9	50.5	Nil Effect	CSII: 2.2 SAP: 0
Chaugule et al. (2017)^19^	46	8.6 ± 0.7	19	47.0	CGM: 1.0 (SD 0.7%) Control: 0.4 (SD 0.7%)	50% reduction
Wan et al. (2018)^20^	Cont^f^.: 51.4 ± 10.9 CGM: 45.7 ± 13.6	Cont.: 8.6 ± 0.6 CGM: 8.6 ± 0.7	Cont.: 23.1 ± 14.5 CGM: 19.6 ± 13.6	Cont: 43.0 CGM: 45.0	N/A	Control: 4% CGM: 2%
Roze et al. (2020)^21^	43 ± 13^d^	8.6 ± 0.6	20 ± 14	44.0	Control: 0.4 CGM: 1	Control: 12.2 CGM: 4.2
	46 ± 13^a,*^	9.1 ± 0.4	20 ± 14	N/A	Control: 0.5 CGM: 1.3	Control: 0 CGM: 3.8
Roze et al. (2021)^22^	47.6 ± 13	8.6 ± 0.6	20 ± 14	44.0	Control: 0.4 CGM: 1	Control: 12.2 CGM: 4.2
Kamble et al. (2012)^23^	41.23 ± 12.19	8.3 ± 0.5	20.23 ± 11.94	43.2	SAP: 1.0 ± 0.7 MDI: 0.4 ± 0.8	N/A
Gomez et al. (2016)^24^	34.19 ± 17.14	9.0 ± 2.0	13.96 ± 9.91	46.5	1.5%	SMBG: 5.22 CGM + CSII: 0.37
Jendle et al. (2019)^25^	37.8 ± 16.5	7.4 (0.9)	21.7	55.6	SAP: 0.5 Control: Nil Effect	HCL: 0 CSII: SHE 1^e^: 65/SHE 2: 25
Pease et al. (2020)^26^	18	8.5	10	53.3	HCL: 0.3	HCL: 0.1^g^ Control: 1.98
Roze et al (2021)^27^	37.8 ± 16.5	7.4 (0.9)	21.7	55.6	SAP: 0.5 Control: Nil Effect	HCL: 0 CSII: SHE 1^e^: 65/SHE 2: 25
Huang et al. (2010)^28^	Cont.: 31.8 ± 17.6^a^ CGM: 29.4 ± 16.3.1^a^	Cont.: 6.50 ± 0.34 CGM: 6.39 ± 0.49	Cont.: 18.15 ± 15 CGM: 16.28 ± 15	Cont.: 52 CGM: 54	CGM: 0.53	Nil effect
	Cont.: 44.7 ± 12.4^c^ CGM: 41.2 ± 11.2^c^	Cont.: 7.61 ± 0.50 CGM: 7.61 ± 0.49	Cont.: 21.83 ± 10 CGM: 23.57 ± 11	Cont.: 57 CGM: 60	Control: gained 0.3% CGM: Maintain HbA1c	Median hypoglycaemia duration CGM: 54 min SMBG: 91 min
McQueen et al. (2011)^29^	40	7.6 ± 0.5%	~20	N/A	0.50%	N/A
Garcia‐Lorenzo et al. (2018)^30^	26	N/A	N/A	N/A	0.23%	Control: 7.9% Intervention: 9.1%

^a^ Scenario 1: Suboptimal glycaemic control (variable definitions HbA1c > 7%, HbA1c > 8.5% * and HbA1c >10% **).

^b^ Scenario 2: At risk of hypoglycaemic events.

^c^ Scenario 3: HbA1c <7% cohort.

^d^ Scenario 4: Cohort aged >25 years.

^e^ SHE 1 requiring non‐medical assistance.

^f^ Cont – control group.

^g^ Study assumed a 95% reduction in rates of SHE.

### 3.3 Description of study results

Table 3. details the cost‐effectiveness of CGM in the form of ICER. The seven studies^12^, ^13^, ^14^, ^15^, ^16^, ^17^, ^18^, ^25^ which compared CSII and SMBG versus SAP and reported a QALY gain within the range of [0.76–2.99] and ICER range of [$18,734–$99,941]. Studies assessing CGM versus SMBG (*n* = 4) in context of MDI reported QALY and ICER gain with the range of [0.54–3.35] and [$19,961– $149,634] respectively. Two studies that compared the combination of MDI and SMBG with SAP, reported QALY gain of [0.376–3.81] and ICERs of [$37,188–$386,667]. Three studies assessed HCL compared to SMBG with CSII or MDI and identified QALY and ICER gains of [1.73–3.72] and [$27,731–$37,767]. For the three studies^28^, ^29^, ^30^ which compared SMBG with CGM regardless of insulin delivery mode, identified QALY gain and ICERs were [0.046–1.11] and [$77,269–$6,019,360] respectively.

**TABLE 3:** Cost‐effectiveness study outcomes and WTP thresholds

Study	Treatment	Location	ICER (Cost per QALY)	ICER (AUD per QALY)	QALY gained	Willingness to Pay Threshold (WTP)	WTP (AUD)^a^	CEAC probability
Roze et al. (2015)^12^	CSII only vs SAP	Sweden	SEK 545,005	$99,942	0.760	SEK 500,000	$91,700	N/A
Roze et al. (2016)^13^	CSII only vs. SAP	France	EUR 30,163	$58,978	1.190	EUR 30,000	$58,700	Hyper: EUR 30,000–80% EUR 50,000–100%
			EUR 22,005	$43,027	1.440			Hypo: EUR 30,000–100%
Roze et al. (2016)^14^	CSII only vs. SAP	United Kingdom	EUR 12, 233	$27,871	2.990	GBP 20,000–30,000	$45,600–$68,400	GBP 20,000–100%
Roze et al. (2017)^15^	CSII only vs. SAP	Denmark	DKK 156,082	$32,538	1.450	DKK 225,000 DKK 375,000	$46,900 –$78,200	Hyper: DKK 225,000–90% DKK 375,000 ‐ >99%
			DKK 89,868	$18,734	1.880			Hypo: DKK 225,000–100%
Conget et al. (2018)^16^	CSII only vs. SAP	Spain	EUR 25,394	$59,833	1.880	EUR 30,000	$70,700	EUR 30,000–97.50%
Nicolucci et al. (2018)^17^	CSII only vs. SAP	Italy	EUR 44,982	$93,772	1.448	EUR 50,000	$104,200	Hyper: EUR 50,000–69.2% EUR 84,000–100%
			EUR 33,692	$70,237	1.877			Hypo: EUR 36,000–68.1% EUR 62,000–100%
Roze et al. (2019)^18^	CSII only vs. SAP	Turkey	TRY 76,971	$65,144	1.400	TRY 80,000	$67,700	Hyper: TRY 80,000–64%, TRY 84,000–80%
			TRY 69,534	$58,850	1.730			Hypo: TRY 80,000–95%
Chaugule et al. (2017)^19^	MDI for both	Canada	CAD 33,78	$43,416	3.350	CAD 50,000	$64,200	CAD 50,000–100%
Wan et al. (2018)^20^	MDI for both	United States of America	USD 98,108	$149,634	0.540	USD 100,000	$152,500	USD 100,000–90%
Roze et al. (2020)^21^	MDI for both	United Kingdom	Euro 9558	$20,130	1.490	GBP 20,000	$42,100	Base case: GBP 20, 000 ‐ ~99%
			Euro 9, 478	$19,961	1.390			Hyper: GBP 20,000–98%
Roze et al. (2021)^22^	MDI for both	Canada	CAD 16,931	$20,188	2.090	CAD 50,000	$59,600	CAD 50,000–100%
Kamble et al. (2012)^23^	MDI vs. SAP	United States of America	3‐day sensor: USD 229,675 6‐day sensor: USD 168,104	$386,667 $283,010	0.376	USD 100,000	$168,400	N/A
Gomez et al. (2016)^24^	MDI vs. SAP	Colombia	USD 24, 000	$37,188	3.810	USD 26,750	$42,500	USD 26,750–99%
Jendle et al. (2019)^25^	CSII only vs. HCL	Sweden	SEK 164,236	$27,731	1.900	SEK 300,000	$50,700	SEK 97.60%
Pease et al. (2020)^26^	MDI vs. HCL	Australia	AUD 37,767	$37,767	3.724	AUD 50,000	$50,000	AUD 50,000–86%
Roze et al (2021)^27^	CSII only vs. HCL	UK	GBP 20,421	$35,942	1.73	GBP 30,000	$62,000	GBP 30,000–99.8%
Huang et al. (2010)^28^	(CSII 90% and MDI 10%)	United States of America	USD 98,679	$172,852	0.60	USD 100,000	$175,200	N/A
			USD 78,943	$138,281	1.11			
McQueen et al. (2011)^29^	CSII or MDI (varied)	United States of America	USD 45,033	$77,269	0.523	USD 100,000	$171,600	70%
Garcia‐Lorenzo et al. (2018)^30^	CSII and MDI	Spain	EUR 2554723	$6,019,360	0.046	EUR 20,000 to 25,000	$47,100–$58,900	~0%

Abbreviations: CEAC, cost effectiveness acceptability curve.

^a^ WTP threshold rounded to the nearest hundred.

Some studies focused on patients with suboptimal management or greater hypoglycaemic risk. In these studies, either benefits from improvement in glycaemic control or reduction in hypoglycaemic risk was assessed, not both. Suboptimal management studies (*n* = 5)^13^, ^15^, ^17^, ^18^ reported an ICER and QALY gain range of [$20,130–$93,772 per QALY] and [1.19–1.45] respectively. Studies analysing patients with greater hypoglycaemic risk (*n* = 5)^13^, ^15^, ^17^, ^18^ reported ICER and QALY gain in the range of [$19,961–$70,236 per QALY] and [1.44–1.88].

Seventeen studies concluded that CGM is cost effective based on willingness‐to‐pay thresholds that ranged from $42,000 to $175,000. Fifteen studies also created a cost effectiveness acceptability curve (CEAC) to help further assess cost‐effectiveness.

## 4 DISCUSSION

The findings of this systematic review support that the implementation of CGM in patients with type 1 diabetes is a cost‐effective strategy: especially in the setting of suboptimal glycaemic control and hypoglycaemic risk. The review also notes that there have been rapid improvements in CGM. This evolution has led to substantial reduction in usage costs and increased accuracy translating to better clinical outcomes.^2^, ^14^, ^31^

### 4.1 CSII

Studies assessing CGM compared to SMBG in the context of CSII, saw narrower ranges in ICERs [$18,734–$99,942] and QALY gained [0.760–2.990] compared to other intervention groups. These studies focused on two subpopulations of type 1 diabetes individuals, those with suboptimal baseline glycaemic management and those at higher risk of hypoglycaemia. Notably, those who were at higher risk of hypoglycaemic events had greater QALY gained and lower ICER compared to those with suboptimal baseline management.

All of the five studies^19^, ^20^, ^22^, ^24^, ^26^ that evaluated individuals with type 1 diabetes at high risk of hypoglycaemic events, based their treatment effects on a single randomized control trial (RCT) by Ly et al.^32^ The patient population selected by Ly et al. were individuals with impaired hypoglycaemic awareness, and had a mean age of 18.6 years, suggesting a significant proportion of paediatric patients. The treatment effect based on a cohort with a paediatric skew, may raise concerns that such effects may not translate to older individuals given youth is a risk factor for hypoglycaemic events.^31^ However, a recent RCT by Richard et al. would quell those concerns, showing a statistically significant reduction in hypoglycemic events with the use of CGM in adults 60 years or older.^2^ It should also be noted that a “low glucose suspend” function was utilized by these SAP however this feature is not employed universally.

Sensitivity analysis from four of the five studies showed, that even if the magnitude of reduction in severe hypoglycaemic events (SHE) by CGM were more than halved, the ICER calculated remained less than the ICER of suboptimal glycaemic management counterparts. This highlights the importance of hypoglycaemic event reduction, as a driver of cost‐effectiveness in CGM.

Five studies^12^, ^13^, ^15^, ^17^, ^18^ also assessed individuals with suboptimal glycaemia management at baseline. These studies had varying baseline HbA1c, ranging from 8.1% to 9%. Four of the studies used the Pickup et al.^4^ meta‐analysis formula to help calculate HbA1c from baseline characteristics. The single other study^12^ based its cohort characteristics on the DCCT study, and its treatment effects from an unpublished meta‐analysis. The treatment effect on HbA1c utilized by this study^12^ was less than what would have been estimated by the Pickup et al.^4^ formula, based on its cohort characteristics.

Of the five studies there were two notably higher ICERs, identified by Roze et al.^12^ and Nicolluci et al.^17^ The high ICER identified by Roze et al.^12^ study can be explained by the noticeably lower QALY gained, almost half that of the other studies. The cause of the lower QALY is unclear as most utility inputs could not be sourced. The higher ICER ($93,772) reported by Nicolluci et al.^17^ may largely be attributed to its high complication treatment cost, which was almost double that identified by the other studies. These five studies, discussed in the previous two paragraphs, highlights how subtle changes in the multitude of inputs required for lifetime modelling, can significantly alter outcomes. These inputs will vary from country to country, and therefore are an important consideration for policy makers when assessing the applicability of these results for their respective countries.

The remaining study by Roze et al.,^14^ which reported the greatest gains in QALYs and lowest ICERs, combined the treatment effects of HbA1c and hypoglycaemic event reduction. Roze et al.^14^ utilized a baseline HbA1c of 10% with a treatment effect of 0.9% HbA1c reduction, as well as a reduction in SHE from 2.2 to 0.0 per patient month. Given an inverse relationship between HbA1c and SHE, it would be prudent to question whether a SHE risk‐reduction of this magnitude would occur in a population with a baseline HbA1c of 10%, noting that the original study had a baseline HbA1c of 7.6%. This study also highlights the paucity of data surrounding real‐world effects of CGM, which is required to accurately evaluate the cost‐effectiveness.

### 4.2 MDI

Currently, four studies^19^, ^20^, ^21^, ^22^ assessed CGM with MDI. Assessment of CGM with MDI remains important, given >65% of patients with type 1 diabetes still use MDI rather than an insulin pump.^33^ Wan et al.^20^ study reported a significantly higher ICER ($149,634) than the other studies despite deeming CGM to be cost‐effective. This was correlated with a noticeably lower QALY gain of 0.54. Chaugule et al.^19^ identified the greatest QALY gain at 3.35, whilst Roze et al.^21^ reported 1.49 and 1.39 QALY gained for its general and suboptimal glycaemic management populations respectively, as well as a QALY gain of 2.09 in the Canadian study.^22^ The variability in the identified ICERs is likely explained by the cost difference of CGM, cost of complications and number of QALY gained.

Additionally, the four studies were based on three different CGM models; Wan et al.^20^ utilized the Dexcom G4, Chaugule et al.^19^ used Dexcom G5 and the two Roze et al.^21^, ^22^ used Dexcom G6. The main impacts of the advancing models include, the introduction of mobile devices being used as receivers from the Dexcom 5 onward, and reduced calibration requirements as well as a longer sensor usage duration with Dexcom 6. As a result, the Chaugule et al.^19^ and Roze et al.^21^, ^22^ studies assumed no or 0.13% of users would require a receiver respectively. This significantly reduced the lifetime costs of CGM, as the original receiver quoted in the Wan et al. study was priced at $737 (USD 482) per year. The cost difference of CGM between the Roze et al.^21^, ^22^ studies and Chaugule et al.^19^ studies also extend from the reduction in transmitter cost and sensor use duration. The cost of transmitters for the Dexcom 5 compared to Dexcom 6 model, as quoted in the studies was approximately $500 and $422 respectively. Additionally, advancement in sensor technology meant the Dexcom 6 sensor used by Roze et al.^21^, ^22^ lasted longer at 10 days whilst, Dexcom 5 (Chaugule et al.^19^) sensors were only used for 7‐days. This resulted in a reduction of 16 sensors required by patients each year, another significant cost reduction. These four studies conducted short periods (2017–2021) apart have already utilized three different model evolutions, highlighting the rapid advancements in CGM technology. The evolving CGM models have also already rapidly cut usage costs, foreshadowing ongoing reductions in CGM ICERs.

### 4.3 HCL

HCL represents a crucial steppingstone to creating a full‐automated artificial pancreas. Of the identified studies only three assessed HCL. All three studies reported low ICERs driven by high QALYs gained. The studies by Jendle et al., Pease et al. and Roze et al. combined the benefits of reduction in HbA1c and reduction in severe hypoglycaemic events. This combined effect reflects evidence that HCL is better able to maintain time within target range and further reduce the risk of hypoglycaemic events compared to SAP.^34^, ^35^

### 4.4 Cost effectiveness assessment

With the rapid discoveries of new treatment and technologies, there has been significant increase in healthcare expenditure. As a result, cost‐effectiveness analysis has become a useful tool for healthcare decision‐makers to help identify treatments that offer larger health gains with less impact on the healthcare budget. Evaluation of therapies and health‐technology are also important for the individual patient, to prevent exposure to “financial toxicity”.^36^ Rapid development of treatments means assessment of cost‐effectiveness are increasingly reliant on outcomes, such as ICER, that are derived from lifetime modelling.

To establish if an intervention is “cost‐effective”, ICERs are compared to a willingness‐to‐pay (WTP) threshold. WTP threshold is defined as “an estimate of what a consumer of health care might be prepared to pay for the health benefit”.^37^

There is no consensus on how best to determine a WTP threshold, which also will vary based on many societal factors. Many countries with a centralized systems of healthcare have WTP threshold to help guide policymakers. Other approaches to identifying WTP thresholds include: (1) per capita income based thresholds, and (2) previous treatment thresholds.^38^ The World Health Organization (WHO) considers an intervention to be cost‐effective if it is three times less than the national annual gross domestic product (GDP) per capita.^39^ Those less than one time the national GDP per capita are considered highly cost‐effective. As for previous treatment thresholds, in the USA, a WTP threshold of AUD ~ $70,000 (USD 50,000) is often quoted.^38^ This was based on the cost of treating end‐stage renal disease when it became enrolled onto Medicare. In this study, we found that limited studies discussed WTP threshold decisions. Two studies utilized the WHO recommended threshold based on GDP per capita, and one study utilized the previous threshold of AUD ~ $70,000. There are additional tools that can be used such as the cost‐effectiveness acceptability curve (CEAC) to support decision makers. The CEAC helps to evaluate the cost‐effectiveness of an intervention by showing the probability that an intervention is considered cost‐effective for a range of monetary values. Thirteen of the reviewed studies also created a CEAC to help further assess cost‐effectiveness.

### 4.5 Outcomes

Two studies (Kamble et al.^23^ and Garcia‐Lorenzo et al.^30^) concurred that CGM was not cost‐effective. These two studies had noticeably higher ICERs at $386,667 (3‐day sensor) and $6,019,360 respectively. There are several areas of contention in these studies. The Kamble et al. study^23^ was performed in 2012 and utilized dated technology. The study itself suggested with further advancements the technology would become more economically attractive Garcia‐Lorenzo et al.^30^ evaluated CGM irrespective of the insulin delivery mode, which likely confounded its assessment, and failed to include the impact of hypoglycaemic events. Additionally, the study created its own Markov Modelling system, which the authors themselves identified as lacking the sophistication of more established models such as the CORE diabetes model. In the model by Garcia‐Lorenzo et al.,^30^ individuals were limited to three concomitant complications and risk factors such as age, other comorbidities and duration of diabetes were not adjusted for. The Garcia‐Lorenzo et al. study highlights the need for robust statistical models to accurately assess cost effectiveness.

Overall, there was acceptance of CGM as a cost‐effective strategy. The majority of ICERs for CGM with MDI was significantly less than three times the GDP per capita of most first world countries. Notably, many were less than one time the GDP per capita of the USA and Australia (as based on data from the World Bank) suggesting CGM is a highly cost‐effective intervention.

### 4.6 Australian context

A recent consensus statement released by the ADS/ADEA/APEG/ADIPS working group highlighted concerns regarding access to diabetes technology.^40^ It identified that high acquisition cost resulted in underutilisation of diabetes management technology, with only 21% of individuals with type 1 diabetes accessing insulin pumps over their lifetime.^41^ Additionally, most of those who accessed this technology did so via private health insurance and were of high‐socioeconomic background. Like insulin pumps, CGM funding remains limited to a select population of individuals. Funding was initially only provided to individuals under 21 years of age, but later expanded to include those intending to conceive or are pregnant, concession cardholders and Aboriginal and Torres Strait Islanders. The limited funding has two main implications. Firstly, most individuals with type 1 diabetes will not qualify for these criteria, thus will not have ready access to a technology that significantly improves clinical outcomes. Resulting in preventable complications and cost being placed on these individuals and society. Secondly, it stands to cause significant distress in individuals who move out of the qualifying categories, putting at risk the progress that was previously made.

Given the similarities between the centralized healthcare systems of Australia and the UK (Medicare and National Health Service), if Australia were to follow in the footsteps of the UK where flash glucose monitoring is funded for all individuals with type 1 diabetes. The benefits experienced in the UK, such as significant reductions in paramedic call‐outs and hospital admissions due to hypoglycaemia and hyperglycaemia, would likely translate.^42^

### 4.7 Limitations

Whilst current cost‐effectiveness studies provide a good basis in guiding healthcare policymakers, limitations remain. As CGM technology improves at rapid rates, there is often a delay in supporting research becoming available. One key example of this is the paucity of studies assessing HCL. It is expected that HCL will be the mainstay version of CGM, yet only three studies were identified in this review. As such, we acknowledge that current on the market CGM devices may be more cost‐effective than what is reported. Additionally, further high qualities studies that minimize confounding factors are needed, with variation in method of treatment being a key confounding factor to be avoided.

We have utilized broad search terms during the review of the available literature to capture greater number or articles, however we acknowledge that there are limitations that come with this. As there are a multitude of different names given to devices under the umbrella term of “continuous glucose monitoring”, if articles had not utilized the umbrella term they may have been missed during the search. The broad nature of CGM also means not all forms (e.g. SAP vs. HCL) under this title are suitable to be compared directly with each other. This is due to the different outcomes and costs associated with each. We have separated the different ways CGM is used in the above discussion to reflect this. Finally, there is also a multitude of different brands of CGM devices which were not all included in this review.

As with any new technology, there is cost associated with widespread implementation. Notably, this includes time spent to help set up and provide support to patients using CGM. Discussion and cost implications surrounding this were absent in all studies. Additionally, CGM provides large volumes of complex data that will require additional time and expertise for a healthcare professional to utilize. Factoring appropriate remuneration for this service was also noticeably absent. These important cost considerations for the successful widespread implementation of CGM will also need to be addressed by healthcare policymakers.

## 5 CONCLUSION

This systematic review provides evidence that CGM appears to be a cost‐effective intervention for individuals with type 1 diabetes. Key drivers of CGM cost‐effectiveness include reduction of chronic complications through improvement in glycaemic management, and reduction in frequency and duration of hypoglycaemic episodes. These studies also highlight the rapidly evolving nature of CGM which has driven down usage costs and may continue to do so with further advances.

## AUTHOR CONTRIBUTIONS

**Yuxin Jiao:** Conceptualization (equal); investigation (equal); methodology (equal); project administration (equal); writing – original draft (equal). **Rose Lin:** Conceptualization (equal); data curation (equal); formal analysis (equal); writing – review and editing (equal). **Xinyang Hua:** Data curation (equal); formal analysis (equal); writing – review and editing (equal). **Leonid Churilov:** Data curation (equal); formal analysis (equal); validation (equal). **Michele J Gaca:** Conceptualization (equal); data curation (equal); writing – review and editing (equal). **Steven James:** Conceptualization (equal); data curation (equal); resources (equal); validation (equal); writing – review and editing (equal). **Philip M Clarke:** Formal analysis (equal); validation (equal). **David Norman O'Neal:** Formal analysis (equal); supervision (equal); writing – review and editing (equal). **Elif I Ekinci:** Conceptualization (equal); project administration (equal); supervision (equal); writing – review and editing (equal).

## FUNDING INFORMATION

No funding was obtained for this article.

## CONFLICT OF INTEREST

EIE is the principal investigators in the NHMRC CTCS funded trial (APP1182464). EIE's institution receives research funding for unrelated research from EliLilly, Boehringer, AstraZeneca, Gilead.

## Data Availability

No further data are available
